# Blob representation of robotic surgical scenes for position-aware video generation

**DOI:** 10.1007/s11548-026-03719-8

**Published:** 2026-06-01

**Authors:** Laurent Mennillo, Chen Katzir, Runlong He, Dimitrios Anastasiou, Danail Stoyanov, Evangelos B. Mazomenos

**Affiliations:** 1 Department of Medical Physics & Biomedical Engineering, University College London, London, UK; 2 Department of Computer Science, University College London, London, UK; 3 UCL Hawkes Institute, University College London, London, UK

**Keywords:** Computer vision for surgical robotics, controllable video generation

## Abstract

**Purpose:**

Synthetic video generation offers substantial promise for AI development in robot-assisted minimally invasive surgery (RMIS), addressing the limited availability of annotated datasets. We present a novel approach for realistic and controllable RMIS video generation, leveraging robot kinematics to constrain the spatial position of the surgical tools in the generated frames.

**Methods:**

Our architecture uses a Blob Generation module that transforms 3D kinematic data into rigid 2D blob representations for spatial conditioning of a Frame Generation module using an encoder–decoder with temporal retention.

**Results:**

Quantitative and qualitative evaluations on the suturing task of the JIGSAWS benchmark dataset demonstrate that our approach enhances both video fidelity and positional accuracy of the surgical tools over long sequences. Blob spatial accuracy against pseudo-ground-truth labels yields average bounding box and mask Dice scores of 0.78 and 0.71, respectively, while video synthesis improves on average by 2% and 5% for SSIM and 4% and 9% for PSNR, while LPIPS reduces by 9% and 20%, compared to baseline state-of-the-art auto-regressive approaches for frames generated at t+10 and t+20.

**Conclusion:**

These results validate the potential of blob representation for position-aware, realistic video generation in RMIS. Synthesized data can augment available datasets, benefiting downstream tasks such as instrument tracking, workflow/skill analysis and robotic automation.

**Supplementary Information:**

The online version contains supplementary material available at https://doi.org/10.1007/s11548-026-03719-8.

## Introduction

Robot-assisted minimally invasive surgery (RMIS) has been widely adopted for a range of surgical procedures [1]. Considerable research efforts have been directed toward the automation of tasks requiring high-level understanding of the surgical scene, such as tool segmentation [2–4], gesture recognition [5, 6], and skill assessment [7–10]. In parallel, considerable progress has been made to increase the level of autonomous operation in RMIS [11–13]. Nevertheless, the limited availability of annotated surgical data poses a substantial challenge for developing and evaluating learning-based models. Various approaches rely on additional information, such as robot kinematics, where available [14], or expanding data distributions through simulation [15].

Image generation techniques focus on synthesizing highly realistic individual images, but have limited applicability in augmenting existing datasets for workflow or skill analysis, which depends on dynamic scene changes and requires understanding and contextualization of human actions across video sequences [16]. Computer graphics simulators [17, 18], explored for RMIS automation, offer flexibility and control but lack the fidelity required for workflow analysis and skill assessment. Furthermore, these frameworks rely on asset-heavy pipelines that demand significant 3D modeling expertise and complex physics rendering.

In recent years, video generation models have seen major advances [19–21]. Most methods rely on high volumes of publicly available datasets to train large and computationally expensive diffusion models that learn to generate realistic and physically plausible video sequences of predefined length. Generation of extended-duration videos is typically addressed through divide and conquer strategies or auto-regressive models [22]. While the former methods initially generate time-space key-frames that are then refined with in-between frames, the latter iteratively predict the next frames based on previously generated data. Auto-regressive approaches are therefore ideal for dynamically controllable video generation, as they allow frame-level control at each iteration.

In the surgical domain, virtual 3D models of surgical tools have been combined with real tissue images to generate realistic-looking, controllable videos [23]. Diffusion models based on textual conditioning, manual input of 2D motion vectors, or segmentation masks have also been employed for the generation of video sequences [24–26], but despite their capacity for high-fidelity synthesis, these methods offer limited control for generating long and complex surgical gestures. Interesting auto-regressive approaches [27, 28], while not aimed at controllable video generation, include a ternary prior-guided variational autoencoder (TPG-VAE) that uses image motion vectors as a prior and samples both content and motion distributions from the latent space of learned surgical gestures, constraining the generation of the next frame to specific classes of gestures.

This work introduces a method for generating controllable RMIS video sequences, enabling visually realistic and domain-accurate data augmentation and simulation for downstream tasks that rely on dynamic temporal changes. Our approach leverages 3D robot kinematics to physically constrain the spatial position of the surgical tools in the generated frames. The proposed two-stage architecture employs a Blob Generation module to transform 3D kinematic data into rigid 2D blob representations, which are then processed for video output by a Frame Generation module using an encoder–decoder architecture with temporal retention. The modularity and iterative nature of our model provide key advantages for simulation applications, where the kinematic data used to constrain tool motion can be easily parametrized to synthesize complex surgical gestures. Moreover, the blobs can be further decomposed and used as grounding representations for additional semantic or structural scene priors, enabling higher fidelity for intricate image details. Our main contributions are: (1) We formulate a novel surgical video generation model conditioned by 3D robot kinematics that can be used both for data augmentation and simulation. (2) We propose a versatile Blob Generation module that learns to map 3D kinematics to the 2D image space in a semi-supervised manner to physically constrain the spatial position of the surgical tools in the generated frames. (3) In experiments on the JIGSAWS dataset, we achieve superior video synthesis compared to baseline state-of-the-art auto-regressive approaches, yielding improvements across evaluation metrics, +2%, +5% for SSIM, +4%, +9% for PSNR, -9%, -20% for LPIPS.

## Methods

Our approach aims to generate a frame $\boldsymbol{\hat{x}_t}$ conditioned by the surgical tool’s kinematic information up to time *t*, given a sequence of past frames $\boldsymbol{x_{1:t-1}}$. In the following sections, the kinematic vectors $\boldsymbol{k_{L, t}}$ and $\boldsymbol{k_{R, t}}$ are 12-element vectors, containing the tooltip 3D coordinates *x*, *y*, *z* and the flattened 3D rotation matrix of the left and right surgical tools, respectively.

### LSTM-AutoEncoder conditioned by robot kinematics

We first evaluated the use of 3D kinematic information for RMIS video generation with a positional conditioning prior using a simple architecture. Inspired by Denton et al. [27], this model is based on a VGG16-like network coupled with an LSTM, keeping skip connections between the encoder and decoder feature layers. Our initial approach, denoted as *LSTM-AutoEncoder* in the subsequent sections, naively treats kinematic data as an extra-dimensional vector added to the encoded features of the input image, by removing the stochastic prior $\boldsymbol{\hat{z}_t}$ in [27] and replacing it with the concatenated kinematics vectors $\boldsymbol{k_t} = [\boldsymbol{k_{L_t}}, \boldsymbol{k_{R_t}}]$. In a second approach, denoted as SVG*, we also evaluated incorporating the kinematic vectors $\boldsymbol{k_t}$ as a stochastic prior, similar to the original architecture. Video generation was performed by auto-regressively inputting previously generated frames.

**Fig. 1:**
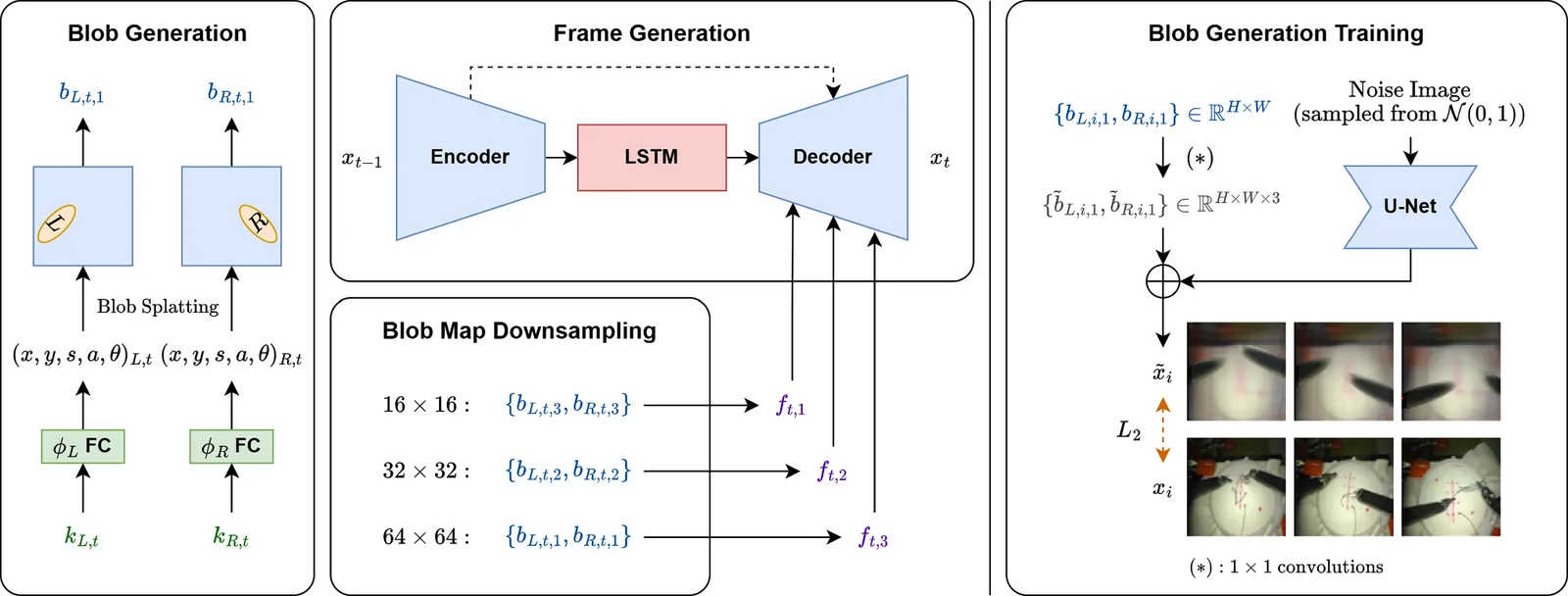
*Left*: Our proposed blob-based video generation model. (i) The Blob Generation module that converts 3D kinematic data to grayscale blob maps. For each frame *i*, the blob maps $\boldsymbol{b_{L, i}}, \boldsymbol{b_{R, i}}$ are generated from the 3D kinematics $\boldsymbol{k_{L, i}}, \boldsymbol{k_{R, i}}$ by splatting the output of the fully connected networks $\phi _{L/R}$. (ii) The Frame Generation module that generates the next frame $\boldsymbol{\hat{x}_t}$ from a given frame $\boldsymbol{x_{t-1}}$ and blob feature maps $\boldsymbol{f_{t, 1}}, \boldsymbol{f_{t, 2}}, \boldsymbol{f_{t, 3}}$. *Right*: Overview of the Blob Generation module’s training scheme. For a given frame *i*, the grayscale blob maps $\boldsymbol{b_{L, i, 1}}, \boldsymbol{b_{R, i, 1}}$ are converted to 3-channel blob maps $\boldsymbol{\tilde{b}_{L, i, 1}}, \boldsymbol{\tilde{b}_{R, i, 1}}$ that are added to a learned background image to generate the predicted image $\boldsymbol{\tilde{x}_i}$. On the right side are examples of frames generated after the training process with their ground-truth counterparts

### Blob-based video generation model

Our proposed blob-based video generation model, inspired by BlobGAN [29] and illustrated in Fig. 1, relies on two key components: **(i)** a Blob Generation module, which transforms 3D kinematic data into rigid 2D representations within the image space, and **(ii)** a Frame Generation module, which produces the next frame in the sequence using an encoder–decoder architecture with temporal retention. The combined framework aims to generate the frame $\boldsymbol{\hat{x}_t}$ given the past conditioning frames $\boldsymbol{x_{1:t-1}}$ and 3D kinematic data up to time *t*, $\boldsymbol{k_{1:t}}$.

#### Blob Generation module

The Blob Generation module transforms 3D kinematic data into rigid 2D representations within the image space using two fully connected (FC) neural networks $\phi _{L}$ and $\phi _{R}$, which convert kinematic vectors ${\boldsymbol{k_{L}}, \boldsymbol{k_{R}}} \in \mathbb {R}^{12}$ to blob representations such that $(x,y,s,a,\theta )_{L/R} = \phi _{L/R}(\boldsymbol{k_{L/R}})$.

An adapted version of the definition proposed in [29] is used to represent 2D blobs in image space. A blob is defined by its center $x, y \in \mathbb {R}$, its scale $s \in \mathbb {R}$, its aspect ratio $a \in \mathbb {R}$, and its rotation $\theta \in [-\pi , \pi ]$. Denoting $\boldsymbol{r}=(x, y)$, blobs are splatted onto a predefined grid $\boldsymbol{r_{{ \textrm{grid}}}} \in \{(\tfrac{w}{W_{grid}}, \tfrac{h}{H_{{ \mathrm {{ \textrm{grid}}}}}})\}$ such that $w, h \in \mathbb {N}$, $w \in [-W_{{ \textrm{grid}}}, W_{{ \textrm{grid}}}]$, $h \in [-H_{{ \textrm{grid}}}, H_{{ \textrm{grid}}}]$, and $W_{{ \textrm{grid}}}$, $H_{{ \textrm{grid}}}$ are the width and height of the grid, respectively. Splatting is done by computing the Mahalanobis distance from every grid point to the blob center:

1 $$\begin{aligned} d(\boldsymbol{r_{{ \textrm{grid}}}}, \boldsymbol{r}, a, \theta ) = (\boldsymbol{r_{{ \textrm{grid}}}} - \boldsymbol{r})^T (R \Sigma R^T)^{-1} (\boldsymbol{r_{{ \textrm{grid}}}} - \boldsymbol{r}) \end{aligned}$$

where $$\Sigma = c \bigl [{\begin{smallmatrix}a &  0 \\ 0 &  \frac{1}{a}\end{smallmatrix}}\bigr ]$$, *R* is a 2D rotation matrix with angle θ and *c* controls the blob edge sharpness. In [29], c=0.02 is used to generate smooth blobs, but since our application requires sharp blobs to accurately represent the surgical tools in image space, we chose c=500 based on empirical inspection of generated blobs. The opacity at a given grid location is given by:

2 $$\begin{aligned} o(\boldsymbol{r_{{ \textrm{grid}}}}, \boldsymbol{r}, s, a, \theta ) = \sigma (s - d(\boldsymbol{r_{{ \textrm{grid}}}}, \boldsymbol{r}, a, \theta )) \end{aligned}$$

with σ the sigmoid function, ensuring that blob map values are capped between (0, 1). Finally, the conversion from kinematics $\boldsymbol{k}$ to grayscale blob maps $\boldsymbol{b}$ is computed as:

3 $$\begin{aligned} \boldsymbol{b}(\boldsymbol{r_{{ \textrm{grid}}}}, \boldsymbol{k_{L/R}}) = o(\boldsymbol{r_{grid}}, \phi _{L/R}(\boldsymbol{k_{L/R}})) \end{aligned}$$

#### Frame Generation module

The Frame Generation module uses a U-Net-like architecture with an LSTM network on the lowest feature level. To generate the next frame $\boldsymbol{\hat{x}_t}$, the previous frame $\boldsymbol{x_{t-1}}$ is given as input to the encoder. The generated blob maps $\boldsymbol{b_{L, t, 1}}$ and $\boldsymbol{b_{R, t, 1}}$ at time *t* are downsampled 2 times by a factor of $\frac{1}{2}$ to create four lower resolution blob maps $\boldsymbol{b_{L, t, 2}}$, $\boldsymbol{b_{R, t, 2}}$, $\boldsymbol{b_{L, t, 3}}$ and $\boldsymbol{b_{R, t, 3}}$. Left and right blob maps are then merged at each resolution and expanded on the feature dimension using 1×1 2D convolutions to create blob feature maps $\boldsymbol{f_{t,1}} \in \mathbb {R}^{H \times W}$, $\boldsymbol{f_{t,2}} \in \mathbb {R}^{\frac{H}{2} \times \frac{W}{2}}$ and $\boldsymbol{f_{t,3}} \in \mathbb {R}^{\frac{H}{4} \times \frac{W}{4}}$. Those are finally concatenated to the corresponding feature maps on the decoder.

### Training procedure

The two modules of our framework are trained consecutively in two separate steps. In the first step, the Blob Generation module trains the networks $\phi _{L/R}$ to generate blob representations from the robot kinematics. This assumes a static camera setup, and this mapping is learned specifically for the camera intrinsics of the training set. In the second step, the networks $\phi _{L/R}$ are frozen to train the Frame Generation module.

#### Blob Generation module

The objective of the $\phi _{L/R}$ networks is to generate blob representations that accurately overlap real surgical tools when splatted on the ground-truth images. Given the absence of ground-truth values for blobs, $\phi _{L/R}$ are trained in the same 3-channel image space as the ground-truth frames, leveraging the pixel intensity difference between the surgical tools and the background. To this end, a background image approximation serving as a global, low-frequency template for the whole training set, is learned from a noise image sampled from $\mathcal {N}(0, 1)$ using a U-Net network. The generated grayscale blob map representations $\boldsymbol{b_{L/R, i, 1}}$ are expanded channel-wise using 1×1 2D convolutions, which are learned during training, and the resulting color blobs $\boldsymbol{\tilde{b}_{L/R, i, 1}}$ are added to the background template to generate the frame $\boldsymbol{\tilde{x}_i}$. The training objective is then to minimize the MSE between the generated frame $\boldsymbol{\tilde{x}_i}$ and the ground-truth frame $\boldsymbol{x_i}$, as depicted in Fig. 1.

#### Frame Generation module

The objective of the Frame Generation module is to predict $N_{\mathrm{{pred}}}$ sequential frames using $N_{\mathrm{{cond}}}$ past conditioning frames while continuously receiving blob feature maps computed from ground-truth kinematics. The output of this module is directly compared to the ground-truth images using image similarity metrics, specifically a combination of mean squared error (MSE) and Perceptual Similarity [30]. Additionally, as auto-regressive models tend to reproduce the last observed frame, motion-based weighting is employed to emphasize high-movement clips during training. Finally, the loss function is defined as:

4 $$\begin{aligned} \mathcal {L}(X) = W(X) \cdot \bigg [\sum ^{T_{tot}}_{t = 1} \textrm{MSE}(\boldsymbol{\hat{x}_t}, \boldsymbol{x_t}) + \gamma \cdot \textrm{PerSim}(\boldsymbol{\hat{x}_t}, \boldsymbol{x_t})\bigg ] \end{aligned}$$

5 $$\begin{aligned} W(X) = \sum ^{T_{tot} - 1}_{t = 1} D(\boldsymbol{p_{L_t}}, \boldsymbol{p_{L_{t+1}}}) + D(\boldsymbol{p_{R_t}}, \boldsymbol{p_{R_{t+1}}}) \end{aligned}$$

such that $p_{L_{t}}$, $p_{L_{t}}$ are Cartesian coordinates of the left and right tools, respectively, D(·) is the Euclidean distance, γ is a weighting factor, and $\textrm{PerSim}(\cdot )$ is the perceptual similarity metric using a pre-trained VGG16 network.

**Table 1:** Blob spatial accuracy (mask and bounding box Dice scores) alongside Spearman rank correlations (ρ) with kinematic metrics (velocity, inter-tool distance and rarity) and geometric accuracy, computed for each LOUO fold

				Mask			BBox	
Fold	Mask Dice	BBox dice	$\rho _v$	$\rho _d$	$\rho _r$	$\rho _v$	$\rho _d$	$\rho _r$
Leave-B-Out	0.69±0.18	0.76±0.14	-0.37	-0.31	-0.44	-0.31	-0.28	-0.44
Leave-C-Out	0.74±0.22	0.84±0.15	-0.50	-0.35	-0.31	-0.48	-0.18	-0.29
Leave-D-Out	0.65±0.21	0.77±0.16	-0.37	-0.46	0.01	-0.43	-0.37	-0.04
Leave-E-Out	0.67±0.17	0.72±0.12	-0.33	-0.26	-0.06	-0.20	-0.35	-0.13
Leave-F-Out	0.75±0.18	0.82±0.13	-0.54	-0.48	-0.56	-0.47	-0.42	-0.38
Leave-G-Out	0.73±0.18	0.82±0.12	-0.35	-0.46	-0.27	-0.12	-0.21	-0.15
Leave-H-Out	0.70±0.22	0.77±0.13	-0.39	-0.49	-0.35	-0.18	-0.19	-0.24
Leave-I-Out	0.72±0.19	0.75±0.14	-0.35	-0.09	-0.42	-0.19	0.07	-0.24
**Overall**	**0.71 ± 0.20**	**0.78 ± 0.14**	**−0.40**	**−0.36**	**−0.30**	**−0.30**	**−0.24**	**−0.24**

The bold values refer to the overall blob spatial accuracy performance and correlation with kinematic metrics (all LOUO folds)

## Experiments

### Dataset and evaluation protocol

We evaluated the proposed methods on the suturing task of the JHU-ISI Gesture and Skill Assessment Working Set (JIGSAWS) dataset [31], which includes synchronized video-kinematic data captured using a da Vinci Surgical System (dVSS). The suturing task is performed on dry laboratory bench-top models and executed five times (trials) by eight participants (subjects) indexed by letters in the interval [**B**, **I**]. The dataset videos were downsampled to 64×64 and 5 Hz from their original resolution and sampling rate (640×480 at 30 Hz) to mitigate compute times. Kinematic data are provided for all frames and contains the tooltips’ Cartesian 3D position (*x*, *y*, *z*) and rotation matrix (*R*) within a common coordinate system for the right and left patient side manipulators (PSM1 and PSM2), respectively, giving 12 degrees of freedom (DOF) for each PSM in total. These kinematic signals inherently contain noise from the robotic system, providing an opportunity to evaluate the model’s robustness on real-world data. For all experiments, we used leave-one-user-out (LOUO) cross-validation as defined in [31] to evaluate the model’s ability to generalize over different participants. The spatial accuracy of the Blob Generation module was evaluated against pseudo-ground-truth instrument masks generated via temporal propagation of manual point prompts using the Segment Anything Model 2 (SAM2) [32]. To account for minor inaccuracies within these zero-shot pseudo-labels, positional accuracy was measured on binarized images of the grayscale blob maps $\boldsymbol{b}$ (with threshold value $\mathrm{{thr}}=127$) using both pixel-wise Dice Similarity Coefficient (DSC) and bounding box DSC. Additionally, we conducted a kinematic correlation analysis using Spearman’s rank correlation coefficient (ρ) to investigate failure cases. Dice scores were correlated against three physical variables on the test sets of each LOUO fold: kinematic linear velocity ($\rho _v$), Euclidean inter-tool distance ($\rho _d$), and spatial kinematic rarity ($\rho _r$), computed via Isolation Forest anomaly scoring [33] on the 24-dimensional kinematic vectors using the implementation provided in [34].

### Implementation details

The $\Phi _{L/R}$ networks of the Blob Generation module are fully connected networks with an input size of 12, an output size of 5, and 3 hidden layers of size 128 with leaky ReLU activation. The encoder part of the Frame Generation module is a VGG16 [35], with input images of size 64 and resulting feature maps of size $512 \times 4 \times 4$, which are then converted using 2D convolutions with a kernel of size 4×4 to vectors of size 256. The LSTM has two layers, with hidden, input, and output sizes of 256. The decoder part is a mirrored version of the encoder, with its pooling layers replaced by spatial up-sampling. Finally, the decoder output is passed through a sigmoid output layer to scale the output to (0, 1). The Blob Generation module was trained using a batch size of 64 single frames, Adam optimizer, learning rate of $\mathrm{{lr}} = 10^{-4}$, and beta parameters set at 0.9 and 0.999. Training was performed for 15 epochs, which showed sufficient convergence for all folds. The Frame Generation module was trained for 20 epochs using Adam optimizer, learning rate of $5 \times 10^{-4}$ and beta parameters set at 0.9 and 0.999. The model was conditioned on $N_{\mathrm{{cond}}} = 10$ frames and trained to predict $N_{\mathrm{{pred}}} = 10$ frames. Movement weighted loss (Eq. 4) was used with $\gamma = 10^4$ and a batch size of 20. As suggested by [27], for all $N_{\mathrm{{pred}}}$ predicted frames, skip connections data of the last conditioning frame were used to improve the model’s ability to retain static background information. Our models were implemented in PyTorch and trained on an NVIDIA RTX 4090 GPU.

## Results

**Fig. 2:**
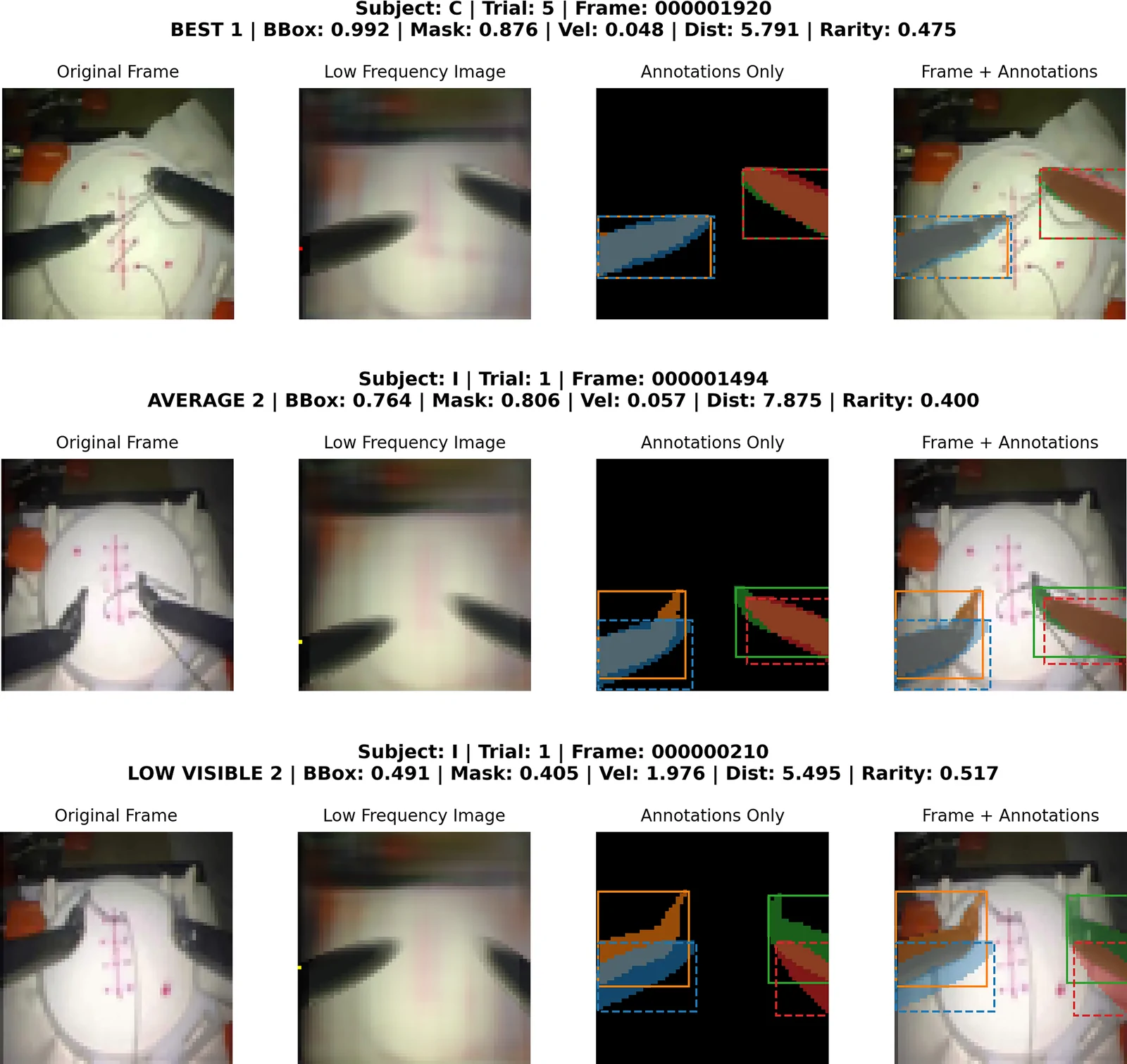
Visual validation of blob positional accuracy. Predicted blob maps $\boldsymbol{b}$ (binarized) and bounding boxes (dashed lines) of the left (blue) and right (red) tools are overlaid against SAM2 ground-truth masks and bounding boxes (solid lines) of the left (orange) and right (green) tools, along with intermediate low-frequency images $\boldsymbol{\tilde{x}}$ illustrating various levels of positional accuracy. One can note the geometric limitation of using elliptical blob representations to accurately overlap highly articulated end-effectors in the middle row, and misalignment of both blobs on the bottom row due to high kinematic velocity. Please refer to the supplementary materials for additional examples

### Blob generation evaluation

#### Positional accuracy

The positional accuracy of the Blob Generation module was evaluated following LOUO cross-validation (Table 1), achieving a global bounding box (BBox) Dice score of 0.78±0.14 (up to 0.84 for the Leave-C-Out fold) and a mask Dice score of 0.71±0.20. These scores demonstrate high spatial accuracy, especially considering the photogrammetric loss (MSE) used for training. Discrepancies arise from a combination of spatial localization errors, inherent SAM2 pseudo-label inaccuracies, and the geometric mismatch between rigid elliptical splats and complex instrument boundaries, especially during extreme end-effector articulations. Because these factors disproportionately penalize pixel-strict metrics, the bounding box DSC serves as a more robust measure of the module’s ability to localize the tools’ functional spatial envelopes. Qualitative samples (with Dice scores) of blob generation are shown in Fig. 2.

#### Failure case analysis

Further analysis of the kinematic correlations (ρ) revealed three distinct causes limiting the module’s performance. First, kinematic velocity exhibited a consistent negative correlation with Dice scores, indicating that high-speed movements induce spatial lag where generated blobs trail the true tool positions. Second, inter-tool distance also correlated negatively with Dice scores, demonstrating that geometric accuracy actually improves as tools converge toward the center of the visual field. This suggests that the Blob Generation module is more accurate during complex, centralized tasks (e.g., needle hand-offs). Finally, correlation scores against kinematic rarity shows a general negative correlation with accuracy, confirming that the network struggles to accurately project rare, out-of-distribution 3D spatial poses into 2D image space.

**Fig. 3:**
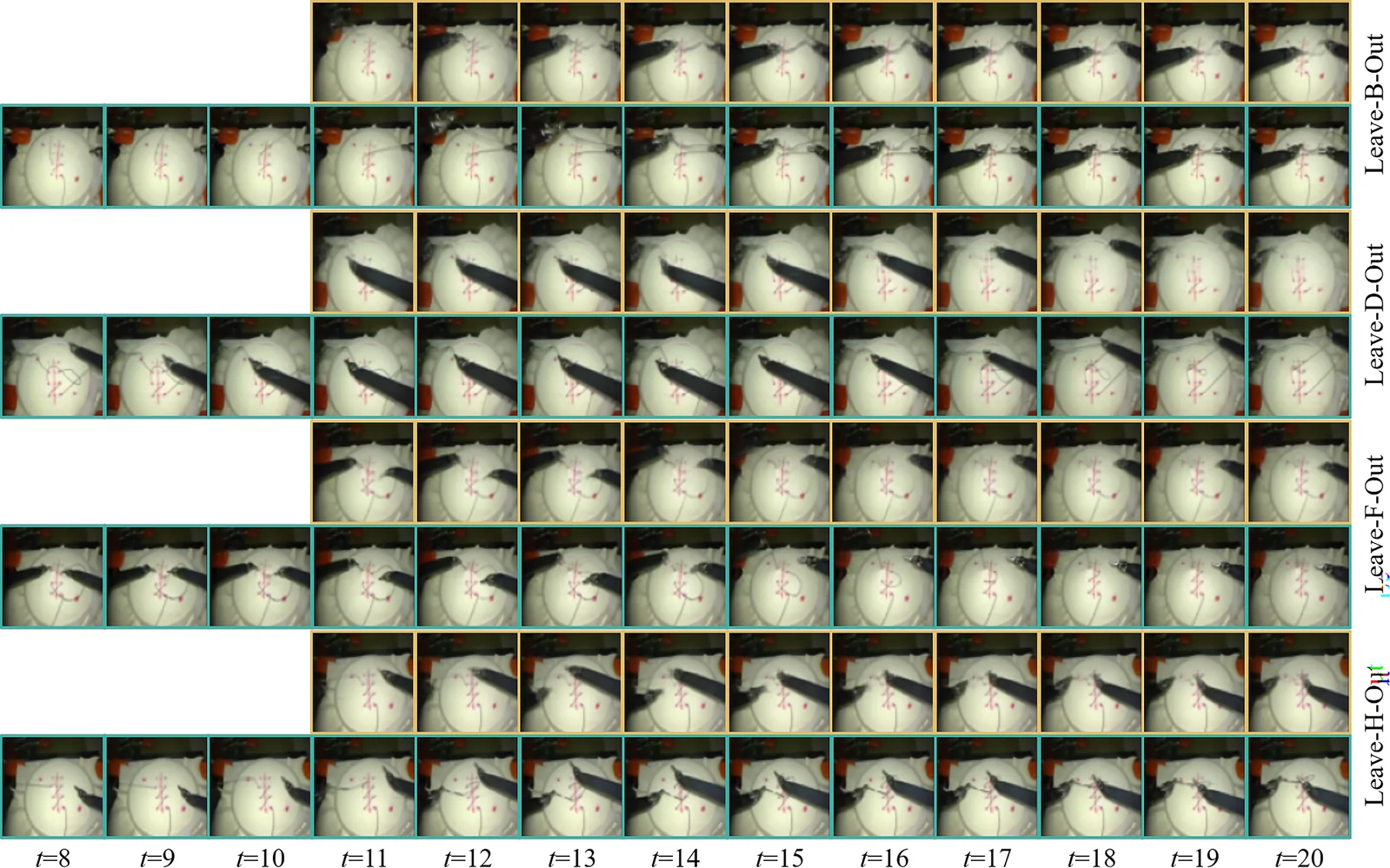
Qualitative results of our blob-based video generation model, showing generated frames from multiple LOUO folds. The top rows in yellow/orange show the generated frames, while the bottom rows in blue/green show the ground-truth. The last three conditioning frames (t=8,9,10) are displayed for visual context, followed by 10 ground-truth and generated frames. Please refer to the supplementary materials for results on the remaining LOUO folds (C, E, G, I)

**Fig. 4:**
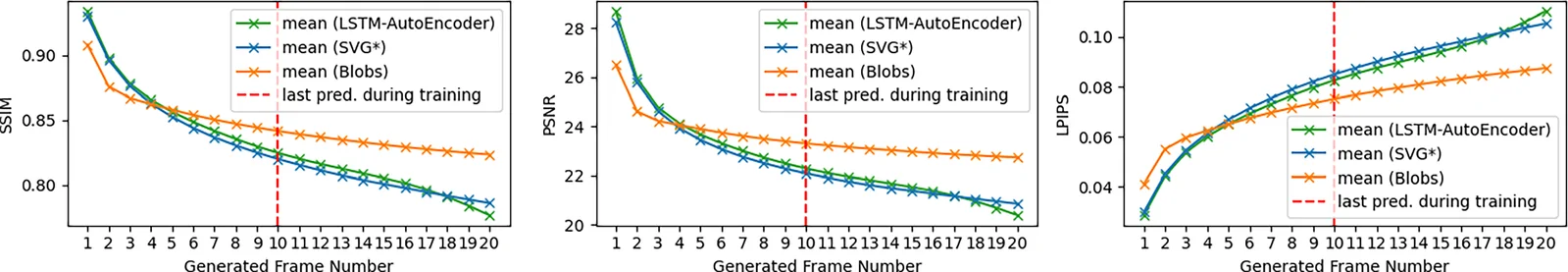
Frame-level similarity metrics for generated frames averaged on all LOUO folds. The x-axis is the sequential index of the generated frame number. The red line marks the last frame index predicted by the model during training

**Table 2:** Mean image similarity metrics computed across all folds

Method	SSIM (↑)	PSNR (↑)	LPIPS (↓)
Frame 10			
Blobs	**0.840 ± 0.036**	**23.212 ± 2.007**	**0.077 ± 0.018**
LSTM-AE	0.823 ± 0.020	22.266 ± 1.085	0.084 ± 0.014
SVG*	0.818 ± 0.023	22.041 ± 1.249	0.086 ± 0.014
Frame 20			
Blobs	**0.822 ± 0.036**	**22.678 ± 1.878**	**0.089 ± 0.019**
LSTM-AE	0.777 ± 0.021	20.452 ± 0.999	0.110 ± 0.014
SVG*	0.784 ± 0.025	20.798 ± 1.181	0.107 ± 0.016

The bold values refer to the best results for each metric at frame 10 and frame 20 (higher SSIM and PSNR values are better, while lower LPIPS values are better)

### Frame generation evaluation

We evaluated our blob-based video generation model against the auto-regressive LSTM-AutoEncoder and SVG* models described in section LSTM-AutoEncoder conditioned by robot kinematics following LOUO cross-validation.

#### Quantitative evaluation

We used the Structural Similarity Index Measure (SSIM), peak signal-to-noise ratio (PSNR), and Learned Perceptual Image Patch Similarity (LPIPS) [30] frame-level similarity metrics to compare the generated images to their ground-truth counterparts and averaged the results according to their sequential index. During training, $N_{\mathrm{{pred}}}=10$ frames were generated. To evaluate the model’s generalizability for longer sequences beyond the training range, we tested the models on an additional 10 frames. As shown in Fig. 4 and Table 2, we can note that the LSTM-AutoEncoder and SVG* models have very similar performance, which is expected since the generation task is deterministic and these models can learn to ignore the stochastic prior. For the first couple of frames, both models have better performance than the blob-based model, which may be attributed to their higher flexibility. However, both aggregate errors quickly fall below the performance of our blob-based model past the third frame.

#### Qualitative evaluation

Figure  3 shows the results of consecutively generated frames for several LOUO folds. The generated images are sharp and accurately capture the static background, despite differences across subjects. The surgical tools are accurately rendered and well-positioned, comparable to the ground-truth images, with minor errors. One observable limitation is the model’s inability to render the needle, string, and suture details. Capturing those fine details would either require integrating dedicated semantic or structural priors for these elements, or integrating the motion dynamics of the whole frame, as proposed in [28], coupled with more advanced generative approaches.

**Fig. 5:**
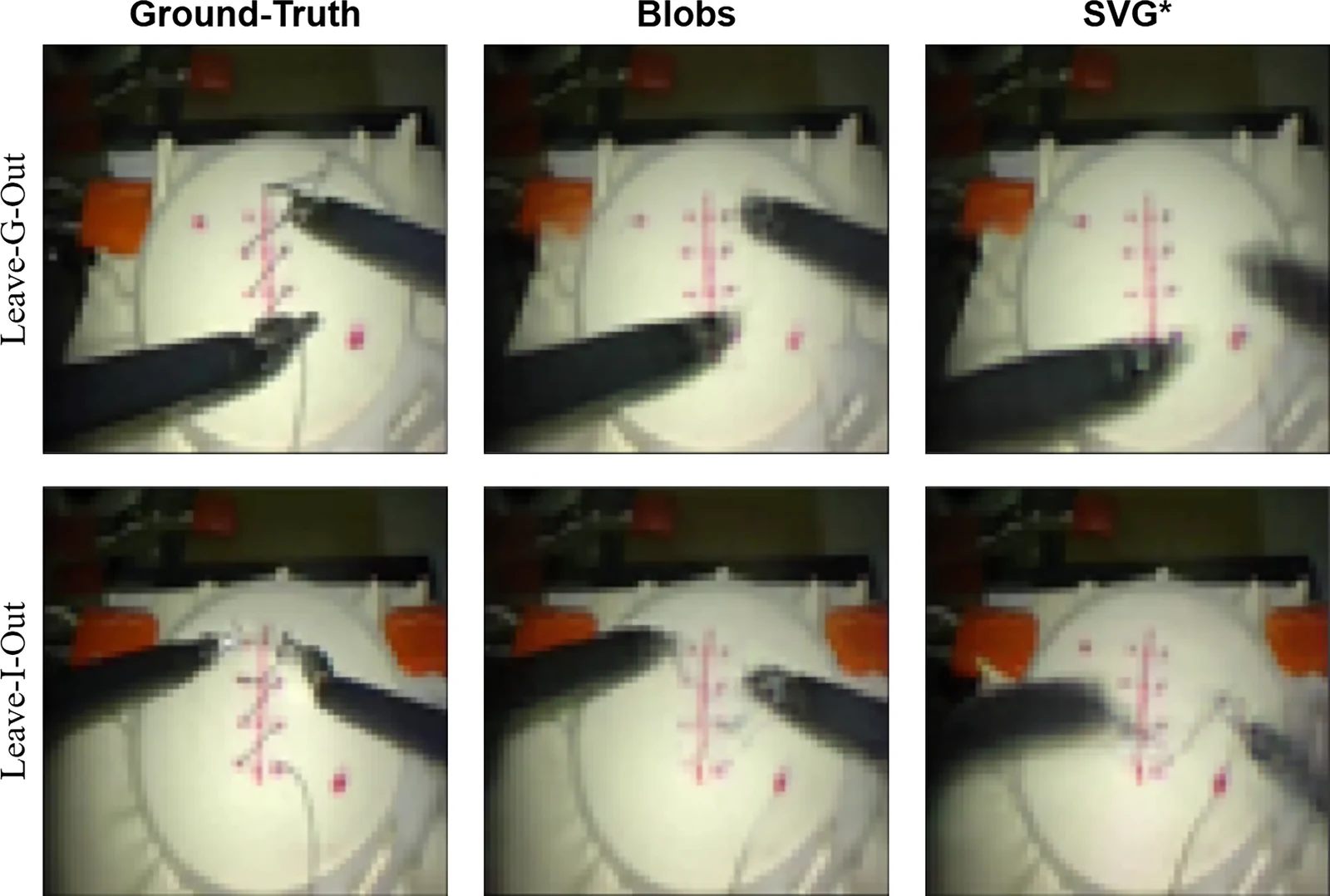
Qualitative results of video generation over long sequences. On the top row, results are shown after 70.2 s. On the bottom row, results are shown after 110.4 s

Figure 5 shows a qualitative comparison of our blob-conditioned model and the SVG* model for frame generation over long sequences. One can note that SVG* generates blurry and deformed instruments, while our model tends to converge toward the generation of rigid, blob-shaped ones.

## Conclusion

This paper presents a novel approach for realistic and controllable RMIS video generation, leveraging robot kinematics to constrain the spatial position of the surgical tools in the generated frames. By incorporating blob representations into the generation process, we demonstrated the ability of our model to generate realistic, position-aware surgical videos that maintain visual consistency across frames.

Through both qualitative and quantitative evaluations, we showed that our model effectively reproduces static backgrounds and accurately positions the surgical tools in the generated frames. Quantitative analysis of the Blob Generation module confirms robust localization, yielding a global bounding box Dice score of 0.78±0.14 and mask Dice score of 0.71±0.20. Furthermore, failure case analysis identified three physical kinematic metrics driving spatial accuracy, demonstrating that while high-velocity movements and rare kinematic poses induce geometric discrepancies, blob positional accuracy peaks during critical, close-proximity surgical gestures. However, qualitative results also show limitations in capturing finer image details such as the needle, string, or sutures, and while our blob-conditioned method outperforms traditional auto-regressive models in maintaining physical consistency of the surgical tools over longer video sequences, further refinement is needed to enhance their rendering for more complex dynamics.

Significant advantages of our method include the ability to parametrize the Blob Generation module to constrain tool motion for synthesis of complex surgical gestures or simulation applications. Furthermore, the modularity of our architecture enables broader integration with state-of-the-art video generation techniques, offering opportunities for further improvements in realism and performance.

Overall, this approach presents a promising direction for enhancing data augmentation and simulation in RMIS for tasks that rely on temporal changes. Future work will focus on increasing the fidelity and resolution of our model on more complex surgical scenes through the integration of diffusion-based generative frameworks and extended blob representations of non-rigid elements, using richer kinematics and additional semantic or structural priors.

## Supplementary Information

Below is the link to the electronic supplementary material.

Supplementary file 1 (pdf 12065 KB)

## Data Availability

The source code and trained models will be made available at: https://github.com/lomenn/Blob_Rep_Video_Generation_RMIS

## References

[1] Kalan S, Chauhan S, Coelho RF, Orvieto MA, Camacho IR, Palmer KJ, Patel VR (2010) History of robotic surgery. J Robot Surg 4(3):141–147. 10.1007/s11701-010-0202-2 27638753

[2] Psychogyios D, Colleoni E, Amsterdam BV, Li C-Y, Huang S-Y, Li Y, Jia F, Zou B, Wang G, Liu Y, Boels M, Huo J, Sparks R, Dasgupta P, Granados A, Ourselin S, Xu M, Wang A, Wu Y, Stoyanov D (2024) SAR-RARP50: Segmentation of surgical instrumentation and Action Recognition on Robot-Assisted Radical Prostatectomy Challenge . https://arxiv.org/abs/2401.00496

[3] Papp D, Elek RN, Haidegger T (2022) Surgical tool segmentation on the jigsaws dataset for autonomous image-based skill assessment. In: International Conference on Computational Cybernetics and Cyber-medical Systems 49–56. 10.1109/ICCC202255925.2022.9922713

[4] Xu J, Wang J, Yu L, Stoyanov D, Jin Y, Mazomenos EB (2025) Personalizing federated instrument segmentation with visual trait priors in robotic surgery. Trans Biomed Eng 72(6):1886–1896. 10.1109/TBME.2025.3526667 40031065

[5] Amsterdam B, Funke I, Edwards E, Speidel S, Collins J, Sridhar A, Kelly J, Clarkson MJ, Stoyanov D (2022) Gesture recognition in robotic surgery with multimodal attention. Trans Med Imaging 41(7):1677–1687. 10.1109/TMI.2022.3147640 PMC7616924 35108200

[6] Shi C, Zheng Y, Fey AM (2022) Recognition and prediction of surgical gestures and trajectories using transformer models in robot-assisted surgery. In: International Conference on Intelligent Robots and Systems. 8017–8024. 10.1109/IROS47612.2022.9981611 PMC10288529 37363719

[7] Anastasiou D, Jin Y, Stoyanov D, Mazomenos E (2023) Keep your eye on the best: contrastive regression transformer for skill assessment in robotic surgery. Robot Autom Lett 8(3):1755–1762. 10.1109/LRA.2023.3242466

[8] Kutana S, Bitner DP, Addison P, Chung PJ, Talamini MA, Filicori F (2022) Objective assessment of robotic surgical skills: review of literature and future directions. Surg Endosc 36(6):3698–3707. 10.1007/s00464-022-09134-9 35229215

[9] Xu J, Sirajudeen N, Boal M, Francis N, Stoyanov D, Mazomenos EB (2025) Sedmamba: enhancing selective state space modelling with bottleneck mechanism and fine-to-coarse temporal fusion for efficient error detection in robot-assisted surgery. Robot Autom Lett 10(1):232–239. 10.1109/LRA.2024.3505818

[10] Shao Z, Xu J, Stoyanov D, Mazomenos EB, Jin Y (2024) Think step by step: chain-of-gesture prompting for error detection in robotic surgical videos. Robot Autom Lett 9(12):11513–11520. 10.1109/LRA.2024.3495452

[11] Schmidgall S, Kim JW, Kuntz A, Ghazi AE, Krieger A (2024) General-purpose foundation models for increased autonomy in robot-assisted surgery. Nat Mach Intell 6(11):1275–1283. 10.1038/s42256-024-00917-4

[12] Attanasio A, Scaglioni B, De Momi E, Fiorini P, Valdastri P (2021) Autonomy in surgical robotics. Annu Rev Control Robot Auton Syst 4:651–679. 10.1146/annurev-control-062420-090543

[13] Yang G-Z, Cambias J, Cleary K, Daimler E, Drake J, Dupont PE, Hata N, Kazanzides P, Martel S, Patel RV, Santos VJ, Taylor RH (2017) Medical robotics—regulatory, ethical, and legal considerations for increasing levels of autonomy. Sci Robot 2(4):8638. 10.1126/scirobotics.aam8638 33157870

[14] Rocha CdC, Padoy N, Rosa B (2019) Self-supervised surgical tool segmentation using kinematic information. In: International Conference on Robotics and Automation, 8720–8726. 10.1109/ICRA.2019.8794334

[15] Colleoni E, Edwards P, Stoyanov D (2020) Synthetic and real inputs for tool segmentation in robotic surgery. In: Medical Image Computing and Computer Assisted Intervention, pp. 700–710. 10.1007/978-3-030-59716-0_67

[16] Boal MWE, Anastasiou D, Tesfai F, Ghamrawi W, Mazomenos E, Curtis N, Collins JW, Sridhar A, Kelly J, Stoyanov D, Francis NK (2024) Evaluation of objective tools and artificial intelligence in robotic surgery technical skills assessment: a systematic review. BJS 111(1):331. 10.1093/bjs/znad331 PMC10771126 37951600

[17] Bendikas R, Modugno V, Kanoulas D, Vasconcelos F, Stoyanov D (2023) Learning needle pick-and-place without expert demonstrations. Robot Autom Lett 8(6):3326–3333. 10.1109/LRA.2023.3266720

[18] Barnoy Y, O’Brien M, Wang W, Hager G (2021) Robotic surgery with lean reinforcement learning. https://arxiv.org/abs/2105.01006

[19] Bar-Tal O, Chefer H, Tov O, Herrmann C, Paiss R, Zada S, Ephrat A, Hur J, Liu G, Raj A, Li Y, Rubinstein M, Michaeli T, Wang O, Sun D, Dekel T, Mosseri I (2024) Lumiere: A space-time diffusion model for video generation. In: SIGGRAPH Asia. 10.1145/3680528.3687614

[20] Ho J, Chan W, Saharia C, Whang J, Gao R, Gritsenko A, Kingma DP, Poole B, Norouzi M, Fleet DJ, Salimans T (2022) Imagen video: high definition video generation with diffusion models. https://arxiv.org/abs/2210.02303

[21] Liu Y, Zhang K, Li Y, Yan Z, Gao C, Chen R, Yuan Z, Huang Y, Sun H, Gao J, He L, Sun L (2024) Sora: A review on background, technology, limitations, and opportunities of large vision models. https://arxiv.org/abs/2402.17177

[22] Li C, Huang D, Lu Z, Xiao Y, Pei Q, Bai L (2024) A survey on long video generation: challenges, methods, and prospects. https://arxiv.org/abs/2403.16407

[23] Ozawa T, Hayashi Y, Oda H, Oda M, Kitasaka T, Takeshita N, Ito M, Mori K (2021) Synthetic laparoscopic video generation for machine learning-based surgical instrument segmentation from real laparoscopic video and virtual surgical instruments. Comput Methods Biomech Biomed Eng Imaging & Vis 9(3):225–232. 10.1080/21681163.2020.1835560

[24] Iliash I, Allmendinger S, Meissen F, Kühl N, Rückert D (2025) Interactive generation of laparoscopic videos with diffusion models. In: DGM4MICCAI – MICCAI Workshop, pp. 109–118. 10.1007/978-3-031-72744-3_11

[25] Cho J, Schmidgall S, Zakka C, Mathur M, Kaur D, Shad R, Hiesinger W (2024) SurGen: Text-Guided Diffusion Model for Surgical Video Generation. https://arxiv.org/abs/2408.14028

[26] Chen T, Yang S, Wang J, Bai L, Ren H, Zhou L (2026) Surgsora: Object-aware diffusion model for controllable surgical video generation. In: Medical image computing and computer assisted intervention, pp. 521–531. 10.1007/978-3-032-05127-1_50

[27] Denton E, Fergus R (2018) Stochastic video generation with a learned prior. In: International Conference on Machine Learning, 80, 1174–1183. https://proceedings.mlr.press/v80/denton18a.html

[28] Gao, X, Jin, Y, Zhao, Z, Dou, Q, Heng, P-A: Future frame prediction for robot-assisted surgery. In: Information Processing in Medical Imaging, 533–544 (2021). 10.1007/978-3-030-78191-0_41

[29] Epstein D, Park T, Zhang R, Shechtman E, Efros AA (2022) Blobgan: Spatially disentangled scene representations. In: European Conference on Computer Vision, pp. 616–635. 10.1007/978-3-031-19784-0_36

[30] Zhang R, Isola P, Efros AA, Shechtman E, Wang O (2018) The unreasonable effectiveness of deep features as a perceptual metric. In: Computer Vision and Pattern Recognition, pp. 586–595 . 10.1109/CVPR.2018.00068

[31] Gao Y, Vedula SS, Reiley CE, Ahmidi N, Varadarajan B, Lin HC, Tao L, Zappella L, Béjar B, Yuh DD, Chen CCG, Vidal R, Khudanpur S, Hager GD (2014) The JHU-ISI gesture and skill assessment working set (JIGSAWS): A surgical activity dataset for human motion modeling. In: M2CAI — MICCAI Workshop

[32] Ravi N, Gabeur V, Hu Y-T, Hu R, Ryali C, Ma T, Khedr H, Rädle R, Rolland C, Gustafson L, Mintun E, Pan J, Alwala KV, Carion N, Wu C-Y, Girshick R, Dollar P, Feichtenhofer C (2025) SAM 2: Segment anything in images and videos. In: International Conference on Learning Representations, vol. 2025, pp. 28085–28128

[33] Liu FT, Ting KM, Zhou Z-H (2008) Isolation forest. In: International Conference on Data Mining, pp. 413–422. 10.1109/ICDM.2008.17

[34] Pedregosa F, Varoquaux G, Gramfort A, Michel V, Thirion B, Grisel O, Blondel M, Prettenhofer P, Weiss R, Dubourg V, Vanderplas J, Passos A, Cournapeau D, Brucher M, Perrot M, Duchesnay É (2011) Scikit-learn: Machine learning in python. J Mach Learn Res 12(85):2825–2830

[35] Simonyan K, Zisserman A (2015) Very deep convolutional networks for large-scale image recognition . https://arxiv.org/abs/1409.1556

