# Supplementary material for: Blob representation of robotic surgical scenes for position-aware video generation
Source: Int J Comput Assist Radiol Surg. 2026 Jun 1;21(7):1485–94. doi: 10.1007/s11548-026-03719-8 (PMC13553727; doi:10.1007/s11548-026-03719-8)
Supplement: Supplementary file 1 [file 11548_2026_3719_MOESM1_ESM.pdf]

Blob Representation of Robotic Surgical Scenes for Position-Aware Video Generation

Laurent Mennillo[1, 3*†], Chen Katzir[1†], Runlong He[1, 3], Dimitrios Anastasiou[1, 3], Danail Stoyanov[2, 3], Evangelos B. Mazomenos[1, 3*]

[1]Dept of Med Phys & Biomedical Eng, University College London, UK.
[2]Dept of Computer Science, University College London, UK.
[3]UCL Hawkes Institute, University College London, UK.

*Corresponding author(s). E-mail(s): l.mennillo@ucl.ac.uk; e.mazomenos@ucl.ac.uk;
Contributing authors: chen.katzir.23@alumni.ucl.ac.uk; runlong.he.23@ucl.ac.uk; dimitrios.anastasiou.21@ucl.ac.uk; danail.stoyanov@ucl.ac.uk;
†These authors contributed equally to this work.

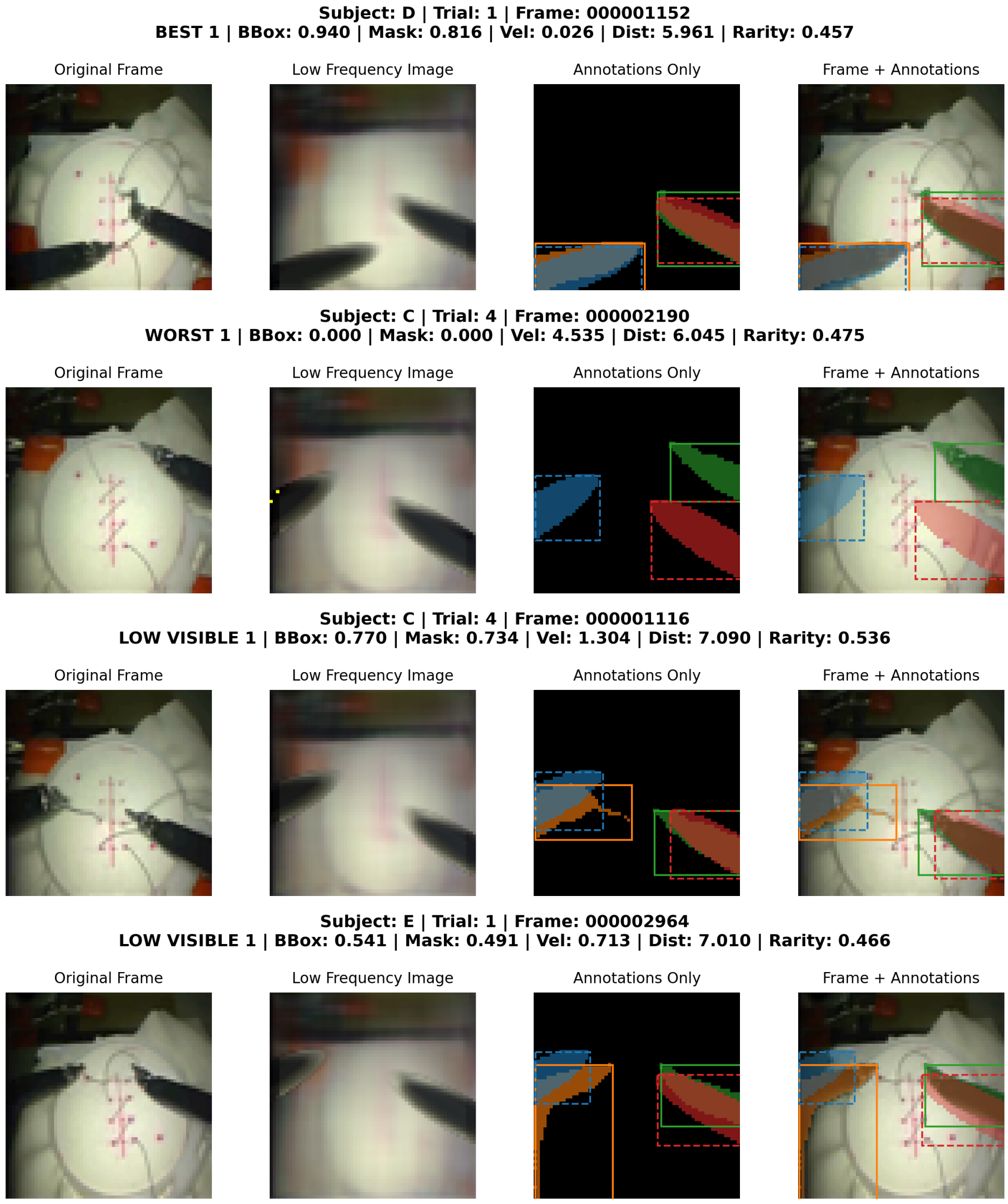

Fig. 1 Visual validation of blob positional accuracy. Predicted blob maps $\boldsymbol{b}$ (binarized) and bounding boxes (dashed lines) of the left (blue) and right (red) tools are overlaid against SAM2 ground-truth masks and bounding boxes (solid lines) of the left (orange) and right (green) tools, along with intermediate low-frequency images $\tilde{\boldsymbol{x}}$, illustrating various levels of positional accuracy. One can note the high impact of kinematic velocity on the first and second rows, leading to extreme Dice scores. Annotation artifacts of the SAM2 pseudo-ground-truth labels are visible for the left instrument in the third and fourth rows (minor and major artifacts, respectively), suggesting that these inherent labeling inaccuracies artificially lower the model's average Dice scores.

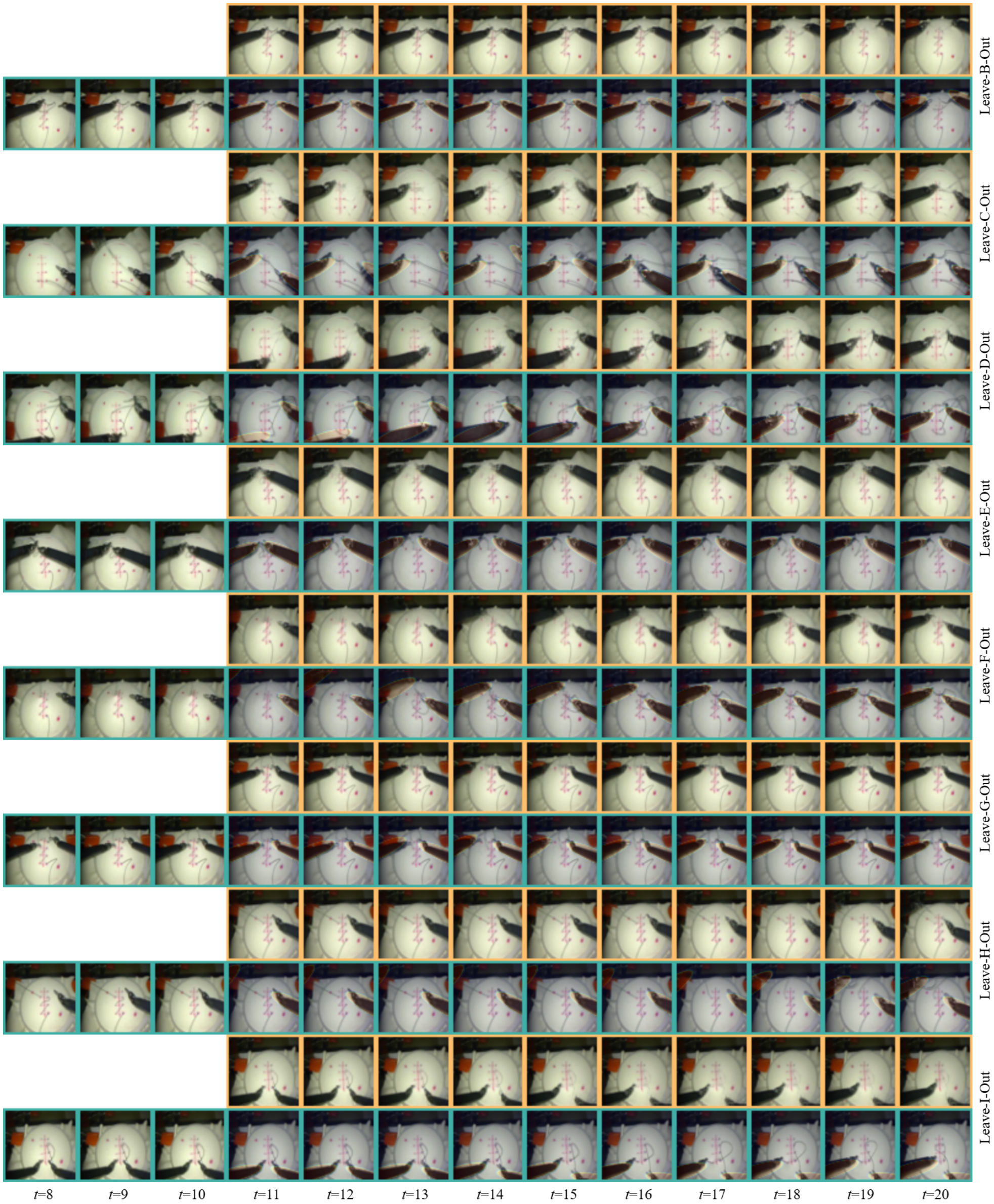

Fig. 2 Qualitative results of our blob-based video generation model, showing generated frames for all LOUO folds with overlayed blobs on the ground-truth images. The top rows in yellow/orange are showing the generated frames, while the bottom rows in blue/green are showing the ground-truth. The last three conditioning frames ($t = 8, 9, 10$) are displayed for visual context, followed by 10 ground-truth and generated frames. Sub-optimal blob positioning can be observed at the end of the sequence on the Leave-B-out fold, on the Leave-D-out fold ($t = 11$ to $t = 13$), and on the Leave-F-out ($t = 13$) fold.

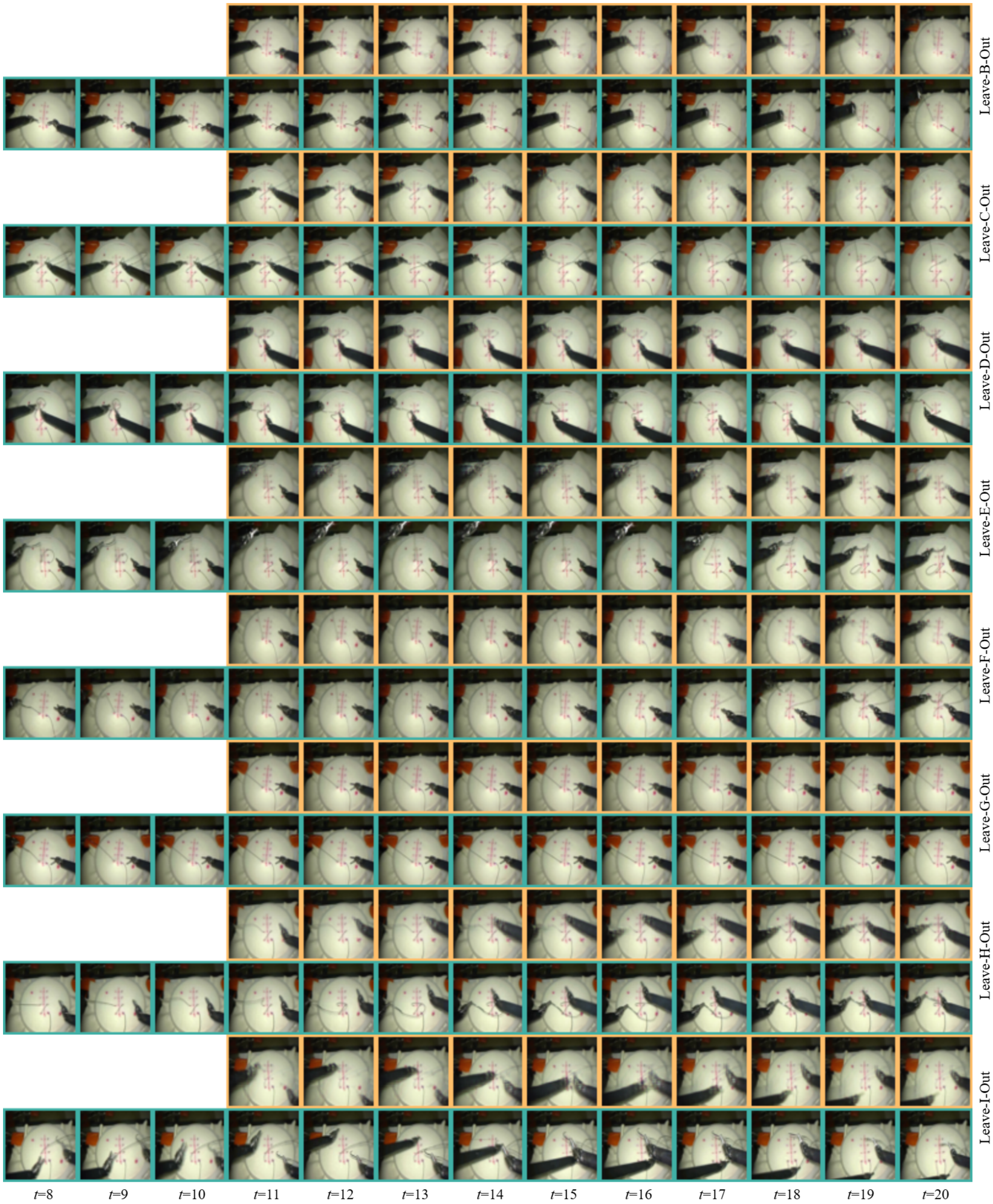

Fig. 3 Qualitative results of the LSTM-AE video generation model, showing generated frames for all LOUO folds. The top rows in yellow/orange are showing the generated frames, while the bottom rows in blue/green are showing the ground-truth. The last three conditioning frames ($t = 8, 9, 10$) are displayed for visual context, followed by 10 ground-truth and generated frames. Towards the end of the sequence in the Leave-D-out fold, the left tool appears to be static in the generated frames, but actually moves in the ground-truth sequence. Additionally, at the end of the Leave-I-out fold, one can notice the tools appear deformed and blurry on the generated frames.

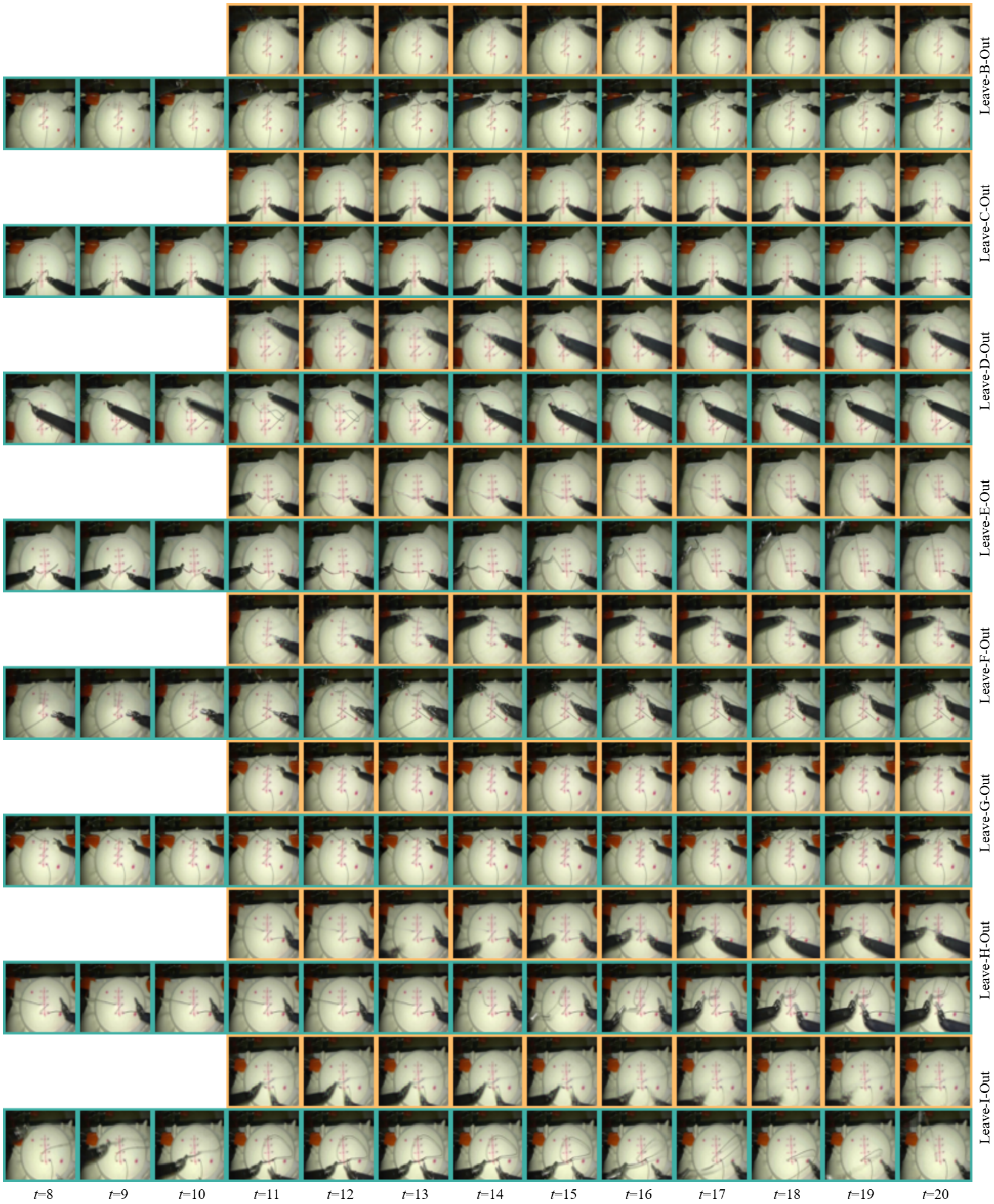

Fig. 4 Qualitative results of the SVG* video generation model, showing generated frames for all LOUO folds. The top rows in yellow/orange are showing the generated frames, while the bottom rows in blue/green are showing the ground-truth. The last three conditioning frames ($t = 8, 9, 10$) are displayed for visual context, followed by 10 ground-truth and generated frames. For most of the generated frames in the Leave-B-out fold, one can notice that the left tool is not rendered at all, while from $t = 13$ to $t = 15$ in the Leave-H-out fold, a left tool is rendered but absent from the ground-truth frames.
